# Factors involved in CLL pathogenesis and cell survival are disrupted by differentiation of CLL B-cells into antibody-secreting cells

**DOI:** 10.18632/oncotarget.3941

**Published:** 2015-05-11

**Authors:** Hussein Ghamlouch, Walaa Darwiche, Ahmed Hodroge, Hakim Ouled-Haddou, Sébastien Dupont, Amrathlal Rabbind Singh, Caroline Guignant, Stéphanie Trudel, Bruno Royer, Brigitte Gubler, Jean-Pierre Marolleau

**Affiliations:** ^1^ EA4666, LNPC, Université de Picardie Jules Verne, Amiens, France; ^2^ Department of Immunology, Amiens University Medical Center, Amiens, France; ^3^ PériTox, Périnatalité & Risques Toxiques, UMR-I 01 Unité mixte INERIS, Amiens, France; ^4^ Department of Molecular Oncobiology, Amiens University Medical Center, Amiens, France; ^5^ Department of Clinical Hematology and Cell Therapy, Amiens University Medical Center, Amiens, France

**Keywords:** chronic lymphocytic leukemia, B-cell differentiation, apoptosis, LEF1, ROR1

## Abstract

Recent research has shown that chronic lymphocytic leukemia (CLL) B-cells display a strong tendency to differentiate into antibody-secreting cells (ASCs) and thus may be amenable to differentiation therapy. However, the effect of this differentiation on factors associated with CLL pathogenesis has not been reported. In the present study, purified CLL B-cells were stimulated to differentiate into ASCs by phorbol myristate acetate or CpG oligodeoxynucleotide, in combination with CD40 ligand and cytokines in a two-step, seven-day culture system. We investigated (i) changes in the immunophenotypic, molecular, functional, morphological features associated with terminal differentiation into ASCs, (ii) the expression of factors involved in CLL pathogenesis, and (iii) the expression of pro- and anti-apoptotic proteins in the differentiated cells. Our results show that differentiated CLL B-cells are able to display the transcriptional program of ASCs. Differentiation leads to depletion of the malignant program and deregulation of the apoptosis/survival balance. Analysis of apoptosis and the cell cycle showed that differentiation is associated with low cell viability and a low rate of cell cycle entry. Our findings shed new light on the potential for differentiation therapy as a part of treatment strategies for CLL.

## INTRODUCTION

Chronic lymphocytic leukemia (CLL) is a heterogeneous disease characterized by clonal proliferation and the accumulation of mature CD5+ B-cells in lymphoid tissues, bone marrow, and peripheral blood. The standard treatment approach is chemoimmunotherapy that leads to significant toxicity and life-threatening immunosuppression, and most patients will relapse [1, 2]. A number of targeted therapies appear to have promise in treating CLL (such as Bruton's tyrosine kinase (BTK) and the delta isoform of phosphoinositol 3-kinase (PI3Kδ) inhibitors [1, 2] and BCL2 family inhibitors [3, 4]). Nevertheless, novel, effective, safe treatment strategies for combination with these agents are still needed for CLL.

Gene expression profiling has been used to characterize CLL-cells and identified several genes whose expression differs between CLL B-cells and normal B-cells including lymphoid enhancer-binding factor 1 (LEF1), receptor tyrosine kinase-like orphan receptor 1 (ROR1), fibromodulin (FMOD), T-cell leukemia/lymphoma 1 (TCL1), Ataxin (ATXN1), early B-cell factor 1 (EBF1) and p27 [5–10] (see also the open web ATLAS (http://amazonia.transcriptome.eu/index.php?zone=Hematology-CLL). LEF1 plays an important role in early normal B-cell differentiation, and is normally expressed in pro-B-cells but not in mature B-cells and plasma cell [11, 12]. LEF1 and ROR1 are expressed in the preleukemic state of monoclonal B-cell lymphocytosis [12, 13] and highly upregulated in CLL B-cells but not normal B-cells and promote leukemic cells growth and survival [12, 14]. TCL1 expression is high in naïve B-cells and absent in memory B-cells and plasma cells [15]. TCL1 was shown to be directly involved in the pathogenesis of CLL and to interact with ROR1 and accelerates development and progression of CLL [13, 16, 17]. FMOD has been found to be highly overexpressed in CLL [6, 18] and its expression is associated with the presence of risk factors [19]. Importantly, it has been shown that LEF1 [11, 12], ROR1 or FMOD knockdown by small interfering RNA induces apoptosis in CLL B-cells [20]. The Cdk inhibitor p27 (a negative regulator of cell cycle progression) is overexpressed in CLL-cells and confers resistance to cell death [21, 22]. Transmembrane activator and calcium modulator and cyclophilin ligand interactor (TACI) (encoded by *TNFRSF13B*) has an important role in B-cell survival, activation, and differentiation [23]. Very recently, it was shown that the prosurvival effect mediated by a proliferation-inducing ligand (APRIL) in CLL B-cells depends on TACI and that the APRIL/TACI interaction significantly accelerates the development of CLL in TCL1 transgenic mice [13, 23]. In CLL, but not in other B-cell malignancies, the BCR was shown to signal autonomously [24]. Pre-existing BCR signaling pathways are critical in the pathogenesis of CLL and have an important role by promoting CLL B-cells survival and proliferation [1, 22, 25, 26]. Furthermore, targeting BCR signaling pathways by siRNA molecules or kinases inhibitors *in vitro* induces downregulation of anti-apoptotic protein myeloid cell leukemia 1 (MCL1) and consequently CLL B-cells apoptosis [26–28]. All these molecules are involved in the pathogenesis of CLL and constitute a part of the malignant program of CLL B-cells [5–10].

The “differentiation therapy” concept for cancer in general requires the development of systems that remove the molecular blocks that prevent malignant cells from maturing into differentiated or normal cells, which no longer grow uncontrollably [29–32]. Thus, reprograming cancer cells to undergo terminal differentiation will result in the loss of proliferative capacity and/or induction of apoptosis [29–32]. Hence, differentiation therapy has been mentioned as a potentially promising way of treating CLL [14, 29, 33–36]. This type of targeted therapy might restore the terminal differentiation program in CLL B-cells and thus avoid the cytotoxicity and complications associated with chemotherapy. Indeed, differentiation therapy has been used successfully in the treatment of acute promyelocytic leukemia [31, 37]. However, successful differentiation therapies for CLL have yet to enter the clinic, despite encouraging results in relatively few preclinical studies [29, 38, 39]. The terminal differentiation of B-cells into antibody-secreting plasma cells is a highly regulated differentiation process that involves profound changes in the B-cells' gene expression profile [40–44] (http://amazonia.transcriptome.eu/index.php?zone=PlasmaCell). We hypothesized that differentiation of CLL B-cells into antibody-secreting cells (ASCs) would be associated with the downregulation of genes involved in the physiopathology of CLL and are expressed (or not) in mature B-cells (e.g. LEF1 and TCL1) but are poorly expressed or not expressed in ASCs.

CLL B-cells are thought to have an arrested B-cell differentiation program. However, there is now renewed interest in studying the differentiation capacity of CLL B-cells [14, 33–36]. Recent research has shown that CLL B-cells display a strong tendency to differentiate into ASCs and may thus be amenable to differentiation therapy [14, 29, 33–35]. In a two-step, 7-day culture system, our laboratory recently demonstrated that phorbol myristate acetate (PMA) and CpG oligodeoxynucleotide induces differentiation of CLL B-cells to an intermediate stage in the plasma cell differentiation process [34, 35]. Using a similar culture systems, in this study we sought to investigate the impact of B-cell differentiation on the expression of factors that contribute to the physiopathology of CLL and/or are known to be deregulated in CLL B-cells (including LEF1, TCL1, ROR1, FMOD, TACI, PI3K, BTK and p27). We also investigated changes in the expression of pro- and anti-apoptotic proteins in ASCs, including MCL1, p53-upregulated modulator of apoptosis (PUMA), X-linked inhibitor of apoptosis protein (XIAP), B-cell lymphoma 2 (BCL2) and B-cell lymphoma-extra-large (BCLxL).

## RESULTS

### 1-Morphologic, immunophenotypic and functional characterization of the resulting ASCs from CLL B-cells synergistically stimulated with PMA and CD40L (PMA/CD40L/c system)

**Figure 1:**
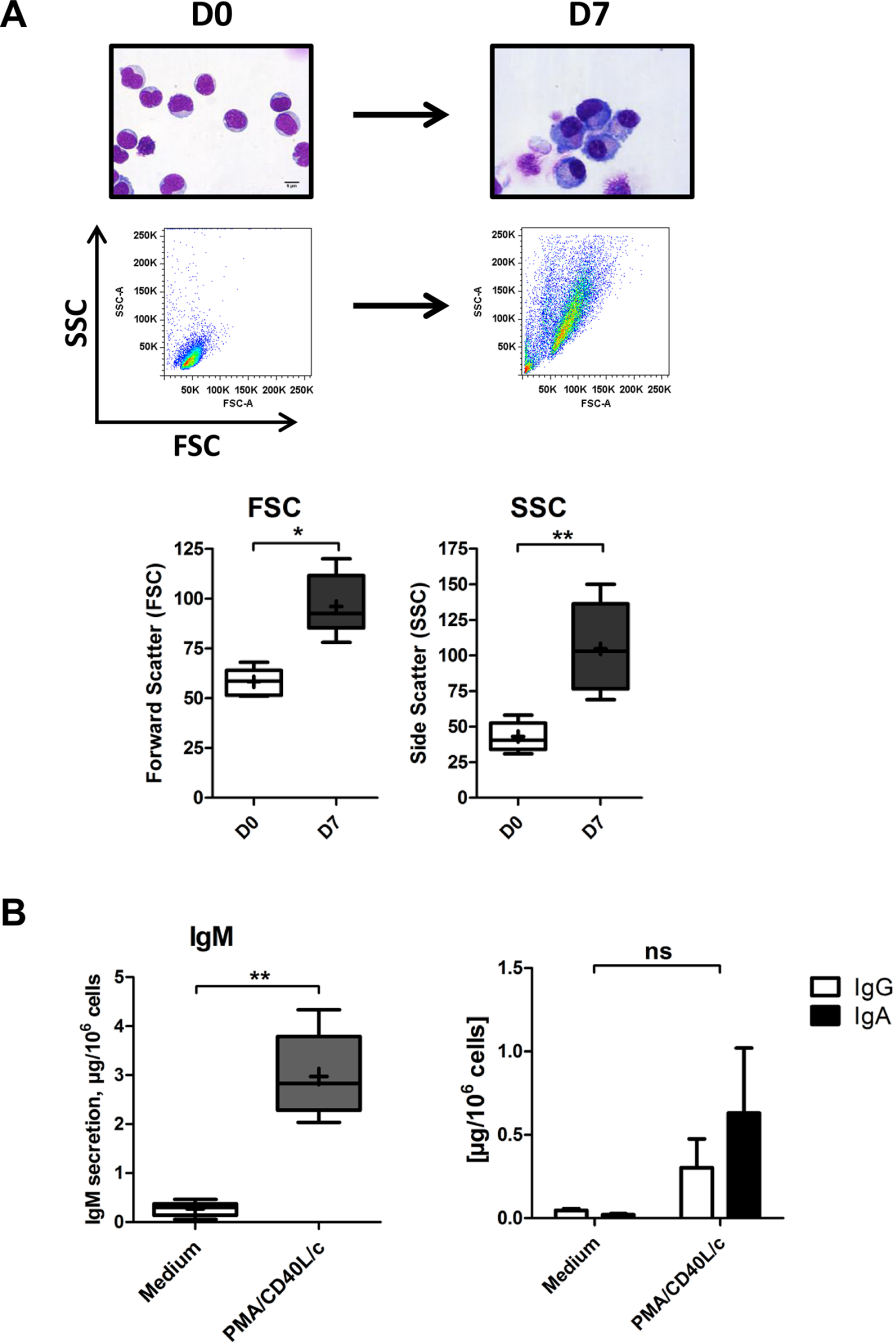
Morphological analysis and Ig secretion On D0, CLL B-cells were stimulated with PMA and CD40L, in combination with the cytokines IL-2, IL-10 and IL-15. On D4, cells were harvested and incubated with IL-2, IL-6, IL-10 and IL-15 for 3 days. **A.** Upper panel: May-Grunwald-Giemsa staining of CLL B-cells on D0 and stimulated cells on D7. Original magnification: x1000. Scale bar, 5 μm. Lower panel: Cell size and granularity were measured by flow cytometry. Relative cell size was determined by assessing the light diffracted at small angles (detected as forward scatter). Granularity is proportional to the light diffracted at large angles (detected as side scatter). Results are represented as box-and-whisker (min to max) plots (the “+” sign indicate the mean). **B.** Culture supernatants were harvested on D7. IgM, IgG and IgA secretion was assessed with an ELISA. The results for six experiments are expressed as box-and-whisker (min to max) plots (the “+” sign indicates the mean (in μg per 10^6^ cells)) for IgM secretion and as the mean ± SEM (in μg per 10^6^ cells) for IgG and IgA secretion. Statistical significance was calculated using the Wilcoxon test: *p < 0.05, **p < 0.01, ns, not significant.

In our previous work, we have characterized in a similar two-step, seven-day culture model the differentiation of CLL B-cells stimulated separately by PMA and CD40L [34]. As CD40L-CD40 interactions and cytokines are important components of the CLL microenvironment, in the present study, we studied the CLL B-cells' ability to differentiate into antibody-secreting plasma cells after stimulation with PMA at the same time as with CD40L. On D0, CLL B-cells were stimulated with PMA and CD40L, in combination with the cytokines IL-2, IL-10 and IL-15. On D4, cells were harvested and incubated with IL-2, IL-6, IL-10 and IL-15 for 3 days. We first investigated the morphological and functional features of the generated ASCs. After seven days of culture in our system, the CLL B-cells acquired an ASC-like morphology, characterized by an eccentric nucleus and well-developed cytoplasm (Figure 1A). These morphological changes were associated with the secretion of large amounts of IgM into the culture supernatant (Figure 1B). IgA and IgG were also detected, albeit at relatively low levels (Figure 1B). These data indicate that CLL B-cells had differentiated into ASCs.

**Figure 2:**
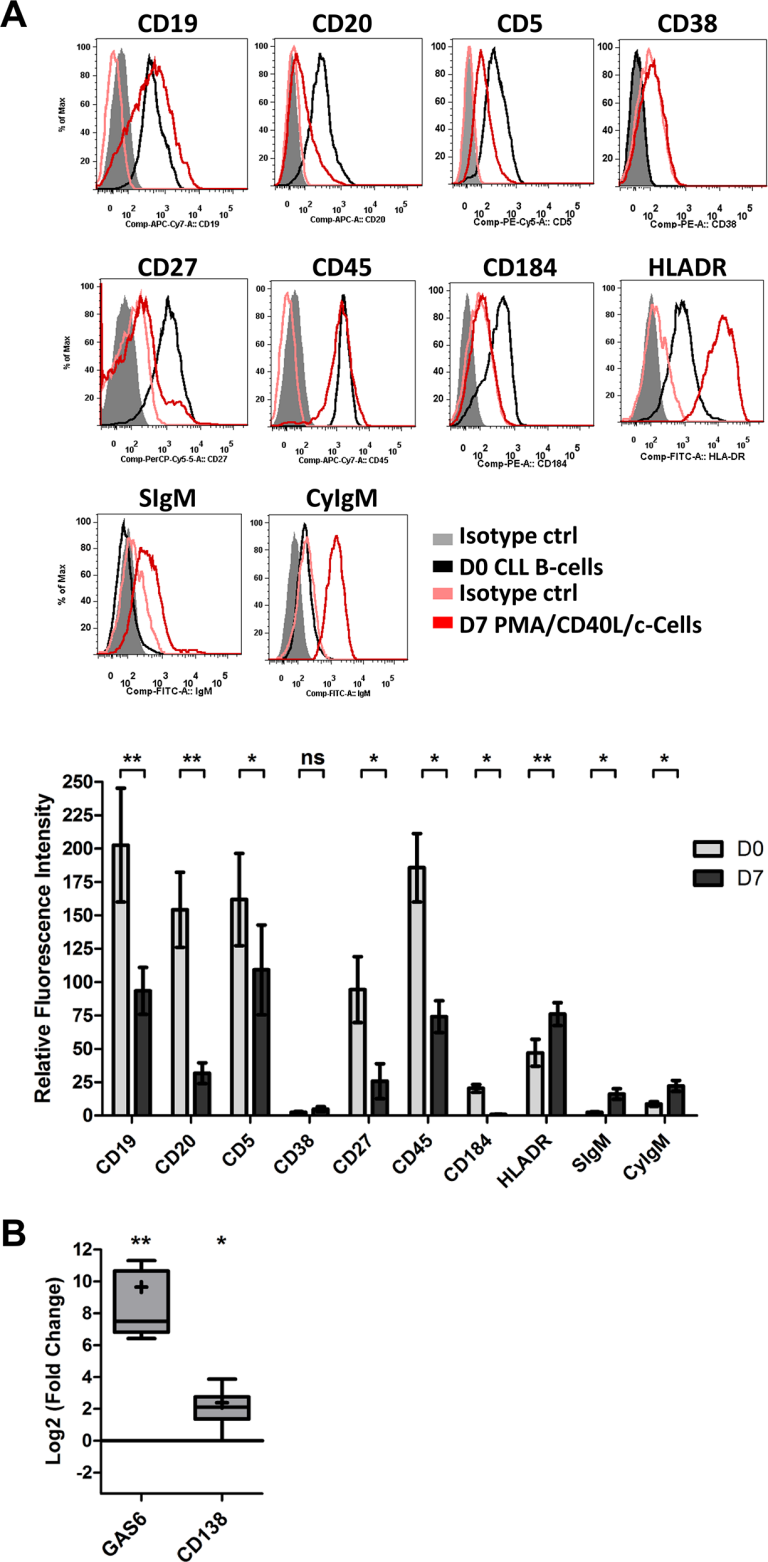
The immunophenotype of the generated ASCs On D0, CLL B-cells were stimulated with PMA and CD40L, in combination with the cytokines IL-2, IL-10 and IL-15. On D4, cells were harvested and incubated with IL-2, IL-6, IL-10 and IL-15 for 3 days. **A.** On D0 and D7, cells were immunophenotyped by direct labeling of CD19, CD20, CD5, CD38, CD27, CD45, CD184, HLA-DR and surface (S)IgM. For cytoplasmic (Cy)IgM, cells were labeled after permeabilization with FITC-conjugated anti-human IgM mAbs or isotype-control mAbs. RFIs were calculated as the ratio of the MFI of cells labeled with a specific Ab to that of cells labeled with a matched isotype control. The Results are represented as mean RFI values ±SEM from eight experiments. Cytometry data are presented as plots for a representative patient. **B.** The expression of the *CD138* and *GAS6* genes was evaluated by quantitative real-time RT-PCR in CLL B-cells on D0 and D7 stimulated cells. Results are expressed relative to gene expression in CLL B-cells on D0, according to the 2^−ΔΔCT^ method. The results are represented as the log2 fold change in box-and-whisker (min to max) plots (the “+” sign indicates the mean) from nine experiments. Statistical significance was calculated using Wilcoxon's test: *p < 0.05, **p < 0.01, ns, not significant.

We next looked at changes in the cell phenotype at D7 (Figure 2A). Consistent with classical plasma cell phenotype, the surface expression of CD19, CD20, CD5 and CD45 was lower for the generated cells than for D0 CLL B-cells (5.4-, 8-, 3.2- and 6-fold, respectively). Plasma cells are characterized by a downregulation or lack of CD20 expression. On D7, 53 ± 22% of ASCs were CD20-negative. The expression of CD27 and CD184 was significantly downregulated (7.2- and 9.6-fold, respectively), whereas the expression of HLADR was significantly upregulated (1.6-fold). The expression of CD38 was not significantly upregulated (2-fold). However, significant upregulation of surface and cytoplasmic IgM expression was observed on D7 (6.4- and 2.6-fold, respectively) (Figure 2A). Furthermore, the cells showed significantly upregulated transcription of the plasma cell marker genes [43] *Gas6* (800-fold) and *CD138* (5.2-fold) on D7 (Figure 2B). CD138 expression was also studied by flow cytometry. However, CD138 was not detected on the cell surface (data not shown). Nevertheless, there were no statistically significant differences between mutated and unmutated CLL samples in terms of morphological features, IgM secretion, immunophenotype and *Gas6* and *CD138* gene expression changes. The fragment analysis and sequencing of the complementarity-determining region 3 of IgH and IgL gene rearrangements (performed on D0 and D7) showed that cells were still clonal after differentiation (Supplementary Figure 1).

### 2-ASCs generated from CLL B-cells display the classical plasma cell transcription program

**Figure 3:**
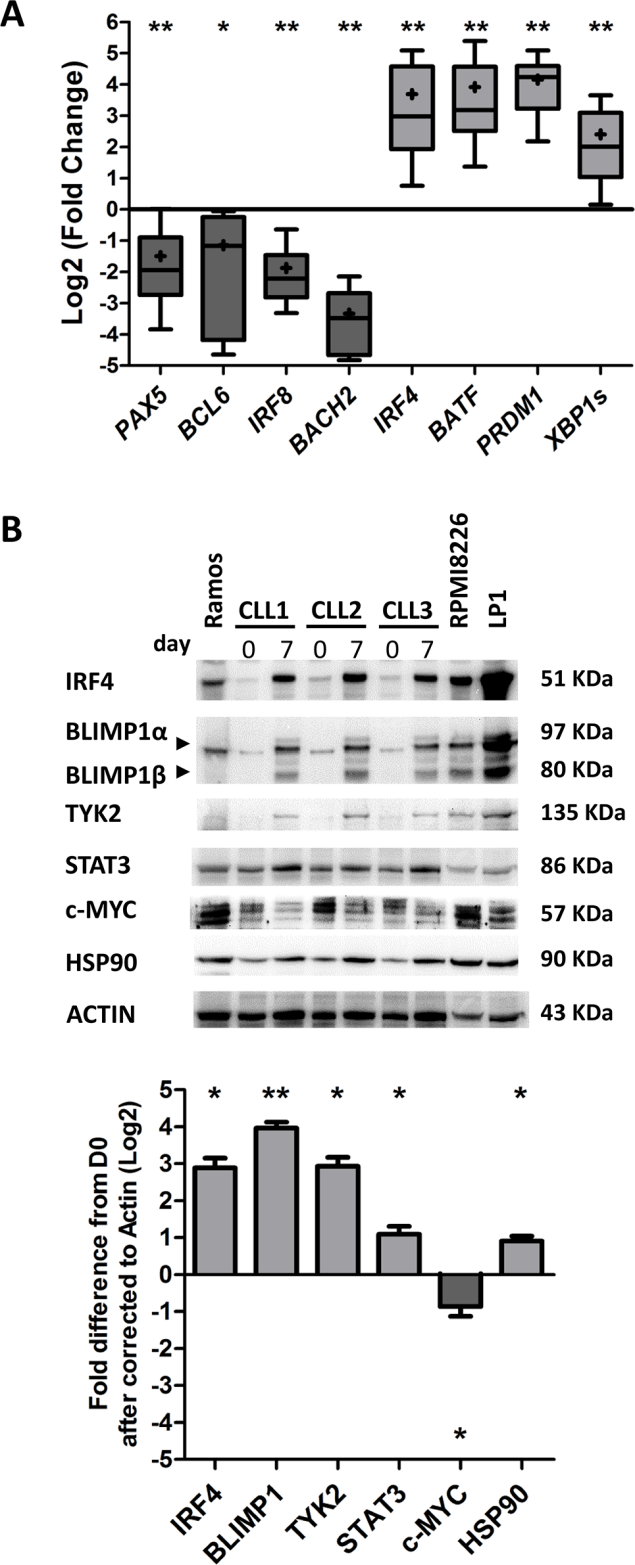
Transcriptional and proteomic analysis of transcription factors involved in plasma cell differentiation **A.** The expression of the *PAX5*, *BCL6*, *IRF8*, *BACH2*, *IRF4*, *BATF*, *PRDM1* and *XBP1s* genes was evaluated by quantitative real-time RT-PCR on D0 and D7. Results are expressed relative to gene expression in CLL B-cells on D0, according to the 2^−ΔΔCT^ method. The results are represented as the log2 fold change in box-and-whisker (min to max) plots (the “+” sign indicates the mean) from 11 experiments. Statistical significance was calculated using Wilcoxon's test: *p < 0.05, **p < 0.01, ns, not significant. **B.** Immunoblot analysis and densitometry quantification of IRF4, BLIMP1, TYK2, STAT3, c-MYC and HSP90 in cells from three CLL samples at D0 and D7. Ramos, RPMI8226 and LP1 cell lines were used as controls. Statistical significance was calculated using Student's *t*-test: *p < 0.05, **p < 0.01.

We next analyzed the molecular mechanisms involved in the terminal differentiation of B-cells into plasma cells in PMA/CD40L/c system. Cells were monitored at D0 and D7 by studying mRNA expression of the B-cell transcription factors *PAX5*, *BCL6*, *IRF8* and *BACH2* (Figure 3A) and the plasma cell transcription factors *IRF4*, Basic leucine zipper transcription factor ATF-like (BATF), *PRDM1*/BLIMP1 and *XBP1s*, by quantitative RT-PCR (Figure 3A). On D7, the transcriptional expression of *PAX5*, *BCL6*, *IRF8* and *BACH2* was significantly downregulated (6.5-, 5.5-, 7.3- and 9-fold respectively), whereas, the transcriptional expression of *IRF4*, *PRDM1*, and *XBP1s* were significantly upregulated (13-, 18- and 5.3-fold respectively) (Figure 3A). The increase in BLIMP1 expression (15.6-fold) (Figure 3B) was confirmed by Western blotting and that of IRF4 (7.4-fold) was confirmed by Western blotting (Figure 3B) and flow cytometry (Supplementary Figure 2). BLIMP1 (the master regulator of plasma cell differentiation) and the spliced form of XBP1 (XBP1s) are involved in the expansion of the ER, the increase in protein synthesis and the upregulation of the unfolded protein response (UPR) [41, 43]. These changes are required for high levels of antibody production and secretion. Recently, it was shown that IRF4 assembles cooperatively with BATF and coordinates the transcriptional program required for the differentiation of peripheral B-cells into ASCs [45, 46]. In our cells, *BATF* expression was significantly upregulated (15-fold) (Figure 3A).

The second step of our differentiation system includes stimulation with cytokines such as IL-6 and IL-10 known to be involved in human ASC differentiation [41, 47, 48]. These cytokines induce the expression of the heat shock protein 90 (HSP90) [49], BATF [50] and the signal transducer and activator of transcription 3 (STAT3), and are also involved in STAT3 activation [51]. The expression of STAT3 and HSP90 is critical for the differentiation and function of ASCs [47, 51, 52]. STAT3 induces BLIMP1 expression [47], which represses the expression of PAX5, BCL6 and c-MYC [47, 51, 53]. STAT3 can bind to the HSP90 promoter and induces its expression [49]. At D7, the expression of STAT3 and its activating tyrosine kinase TYK2 [54] and HSP90 was clearly upregulated in generated-ASC (2.2-, 7.6- and 1.9-fold, respectively) whereas the expression of c-MYC was clearly downregulated (4.5-fold) (Figure 3B). Thus, by analogy with the terminal differentiation program of normal B-cells into plasma cells, CLL B-cells increased their STAT3, IRF4, XBP1s and BLIMP1 expression and decreased their c-MYC, PAX5, BCL6, IRF8 and BACH2 expression. These findings correlates with our previous results and other literature data [14, 33–36], suggesting that CLL B-cells (i) are able to restore the transcriptional program associated with plasma cell differentiation if appropriate stimulation is provided [34–36] and (ii) display relevant ASC features, including morphological changes, UPR induction [34] and initiation of secretory function.

### 3-Differentiation of CLL B-cells induces changes in the expression of CLL-pathogenesis-associated factors

We next investigated the effect of CLL B-cell differentiation in PMA/CD40L/c system on the expression of factors associated with CLL pathogenesis, including LEF1, TCL1, ROR1, FMOD, *TNFRSF13B*/TACI, *BIRC5*/survivin [55], p27, PI3K and BTK. Furthermore, we also measured expression of factors that are deregulated in CLL but that are not known to be directly involved in the pathogenesis of CLL (including Ataxin (ATXN1) [6, 7], *FCER2*/CD23 [6, 7], early B-cell factor 1 (EBF1) [7], myristoylated alanine-rich protein kinase C substrate (MARCKS) [8] and *Ly9*/CD229 [56].

**Figure 4:**
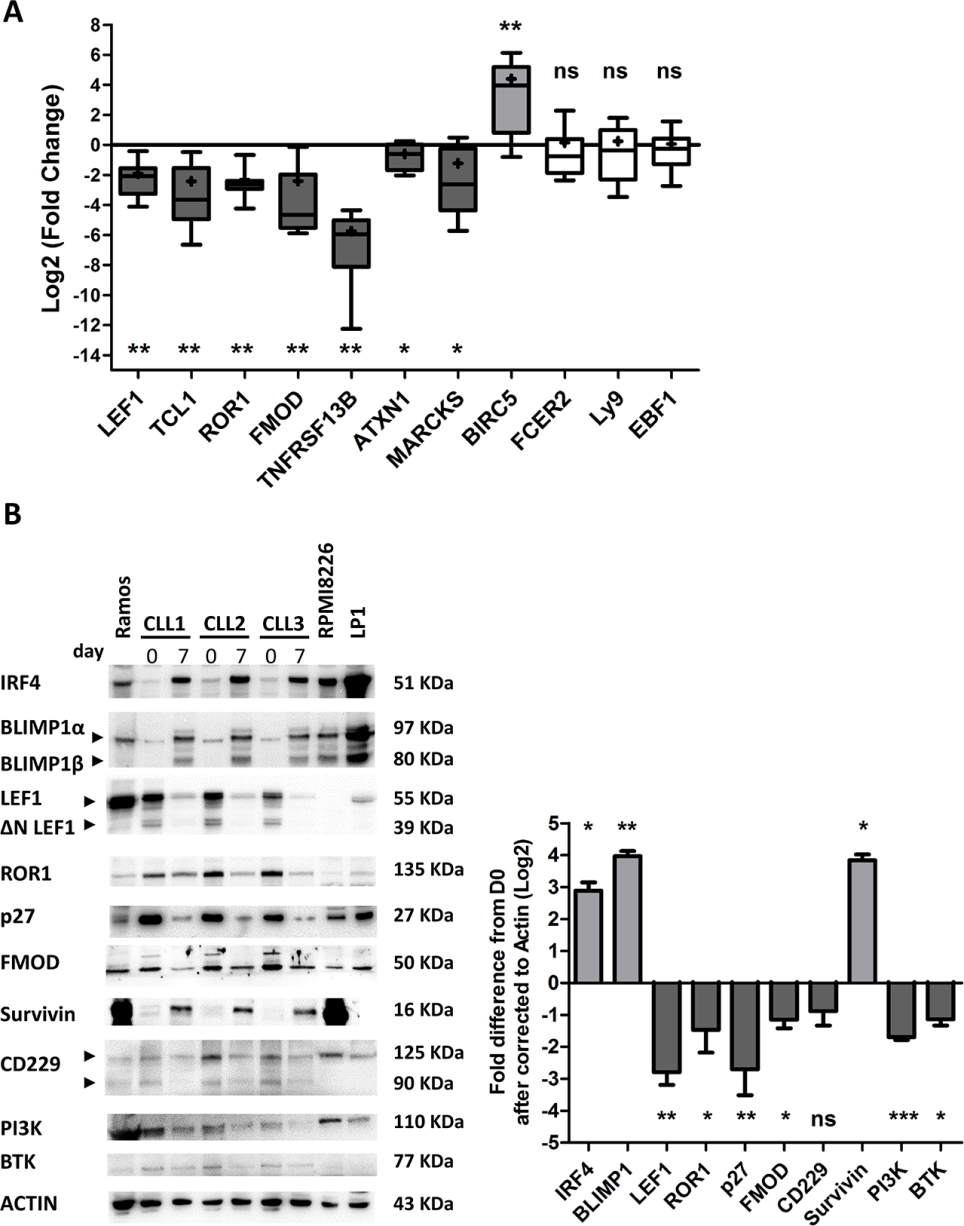
Transcriptional and proteomic analysis of factors involved in CLL pathogenesis **A.** The expression of the *LEF1*, *TCL1*, *ROR1*, *FMOD*, *TNFRSF13B*, *ATXN1*, *MARCKS*, *BIRC5*, *FCER2*, *Ly9* and *EBF1* genes was evaluated by quantitative real-time RT-PCR on D0 and D7. Results are expressed relative to gene expression in CLL B-cells on D0, according to the 2^−ΔΔCT^ method. The results are represented as log2 fold changes in box-and-whisker (min to max) plots (the “+” sign indicates the mean) for 11 experiments. Statistical significance was calculated using the Wilcoxon's test: *p < 0.05, **p < 0.01, ns, not significant. **B.** Immunoblot analysis and densitometry quantification of IRF4, BLIMP1, LEF1 full length and ΔN LEF-1 isoforms, ROR1, p27, FMOD, survivin, CD229, PI3K and BTK in cells from three CLL samples at D0 and D7. Ramos, RPMI8226 and LP1 cell lines were used as controls. Statistical significance was calculated using Student's *t*-test: *p < 0.05, **p < 0.01, ***p < 0.001.

Quantitative RT-PCRs showed that differentiation of CLL B-cells into ASCs induces significant downregulation of *LEF1* (7.4-fold), *TCL1* (8.2-fold), *ROR1* (8-fold), *TNFRSF13B*/TACI (9.8-fold) and *FMOD* (8.1-fold) (Figure 4A). In contrast, *BIRC5*/survivin expression was significantly induced (21-fold) (Figure 4A). However, there was no significant effect on the expression of *FCER2*, *Ly9*/CD229 and *EBF1* (Figure 4A). Immunoblot results confirmed the qRT-PCR data for downregulation of LEF1 (8.6-fold), ROR1 (6.3-fold), FMOD (5.5-fold), and upregulation of survivin (14-fold), and evidenced downregulation of p27 (8.5-fold), PI3K (9.9-fold) and BTK (5.4-fold) (Figure 4B). These observations suggest that the differentiation of CLL B-cells into ASCs is associated with downregulated expression of CLL-pathogenesis-associated proteins, including LEF1, TCL1, ROR1, FMOD, *TNFRSF13B*, PI3K, BTK and p27.

### 4- Differentiation of CLL B-cells into ASCs in PMA/CD40L/c system is associated with incidence of apoptosis but not with exaggerated cell proliferation

We next examined the cell cycle distribution and apoptosis of cells *in vitro*. Indeed, normal B-cell differentiation gives rise to both short-lived and long-lived ASCs [42, 44, 53]. Long-lived ASCs reside in the bone marrow, where survival signals are provided by the environment and maintain long-term antibody production. Short-lived ASCs are rapidly formed from extrafollicular foci in secondary lymphoid organs, where they undergo apoptosis after a few days of intensive antibody secretion (mainly of low-affinity IgM Abs but also isotype-switched Abs). During plasma cell differentiation, the accumulation of misfolded proteins (due to the synthesis of large amounts of antibodies) leads to increase in ER stress. Failure of the UPR to reduce the load of unfolded proteins leads to excessive ER stress followed by cell death. Furthermore, plasma cell differentiation requires the regulation of proliferation and is probably associated with irrevocable cell cycle exit [47, 51, 53]. Indeed, short-lived ASCs die soon after completing differentiation and exiting cell cycle [44, 53, 57].

**Figure 5:**
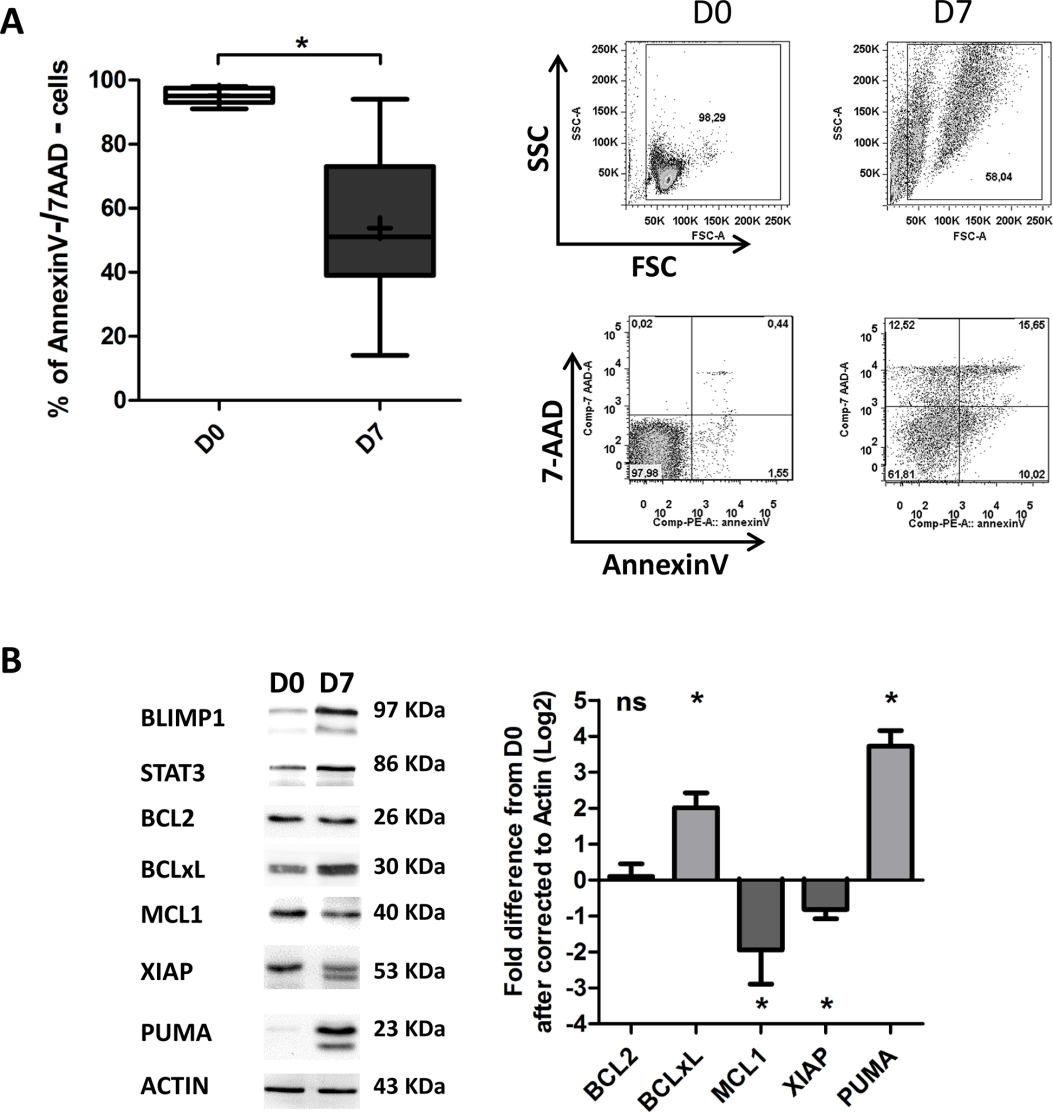
Differentiated CLL B-cells display decreased survival **A.** Cells were stained with Annexin V-PE and 7AAD at D0 and D7 to evaluate apoptosis. Left panel: the percentages of double-negative (i.e. annexin-V-negative and 7AAD-negative) living cells in nine experiments are represented in box-and-whisker (min to max) plots (the “+” sign indicates the mean). Right panel: cytometry plots from a representative patient. **B.** Immunoblot analysis and densitometry quantification of BCL2, BCLxL, MCL1, XIAP and PUMA in cells at D0 and D7. The PUMA and XIAP antibodies also cross-reacts with an 18 kDa band and 47 kDa band respectively of unknown origin. The data shown are representative of three experiments. Statistical significance was calculated using Student's *t*-test: *p < 0.05.

On D7, an Annexin-V/7AAD survival assay revealed apoptosis among the generated ASCs (53 ± 24% of the cells had survived, on average; Figure 5A). Importantly, it was shown very recently that the balance between pro-survival and pro-apoptotic proteins is perturbed during ASC differentiation [4, 40]. Specifically, expression of anti-apoptotic proteins (including BCL2 and MCL1) is downregulated, and expression of pro-apoptotic proteins is upregulated [4]. These changes lead to a reduction in the cell's apoptotic threshold [4]. However, the researchers also showed that during ASC differentiation, cells are saved from differentiation-associated death signals by BCLxL upregulation [4, 40]. We therefore examined the expression of BCL2, BCLxL, MCL1, XIAP and PUMA in the generated ASCs. As shown in Figure 5B, no changes in the expression of BCL2 were detected, whereas downregulation of MCL1 and XIAP (7.4-fold and 4.3-fold, respectively) and upregulation of BCLxL and PUMA (4-fold and 13-fold, respectively) were observed. In order to determine whether the changes in expression of BCLxL and PUMA were specifically related to the differentiation process, we investigated their expression in non-stimulated cells (i.e. medium only). In contrast to differentiated cells, we observed clear downregulation of BCLxL expression and slight upregulation of PUMA expression in non-stimulated cells (Supplementary Figure 3).

Importantly, PUMA was recently shown to be involved in ER-stress induced apoptosis and to regulate the maintenance of XBP1 mRNA splicing [58]. Indeed, the downregulation of MCL1 and XIAP and the upregulation of PUMA in the generated-ASCs suggest that cell death is associated with the differentiation of CLL B-cells. Nevertheless, recent studies suggest that BCLxL promotes the survival of recently generated/short-lived ASCs [4, 57], whereas high expression of MCL1 is needed to promote the survival of long-lived ASCs [43, 48, 57]. Furthermore, ASCs generated in this work, dramatically reduced the expression of *TNFRSF13B*/TACI and CXCR4/CD184 (Figure 2A and Figure 4A) that are critical for the survival of human long-lived ASCs [48, 57]. On this basis, we conclude that the ASCs generated in our culture system are short-lived ASC. However, the downregulation of MCL1 could also be explained by the decreased expression of Wnt pathway molecules (LEF1 and ROR1), TCL1, BCR signaling molecules (PI3K and BTK) and c-MYC that were shown to positively regulate MCL1 expression to promote CLL B-cells survival [11, 14, 28].

**Figure 6:**
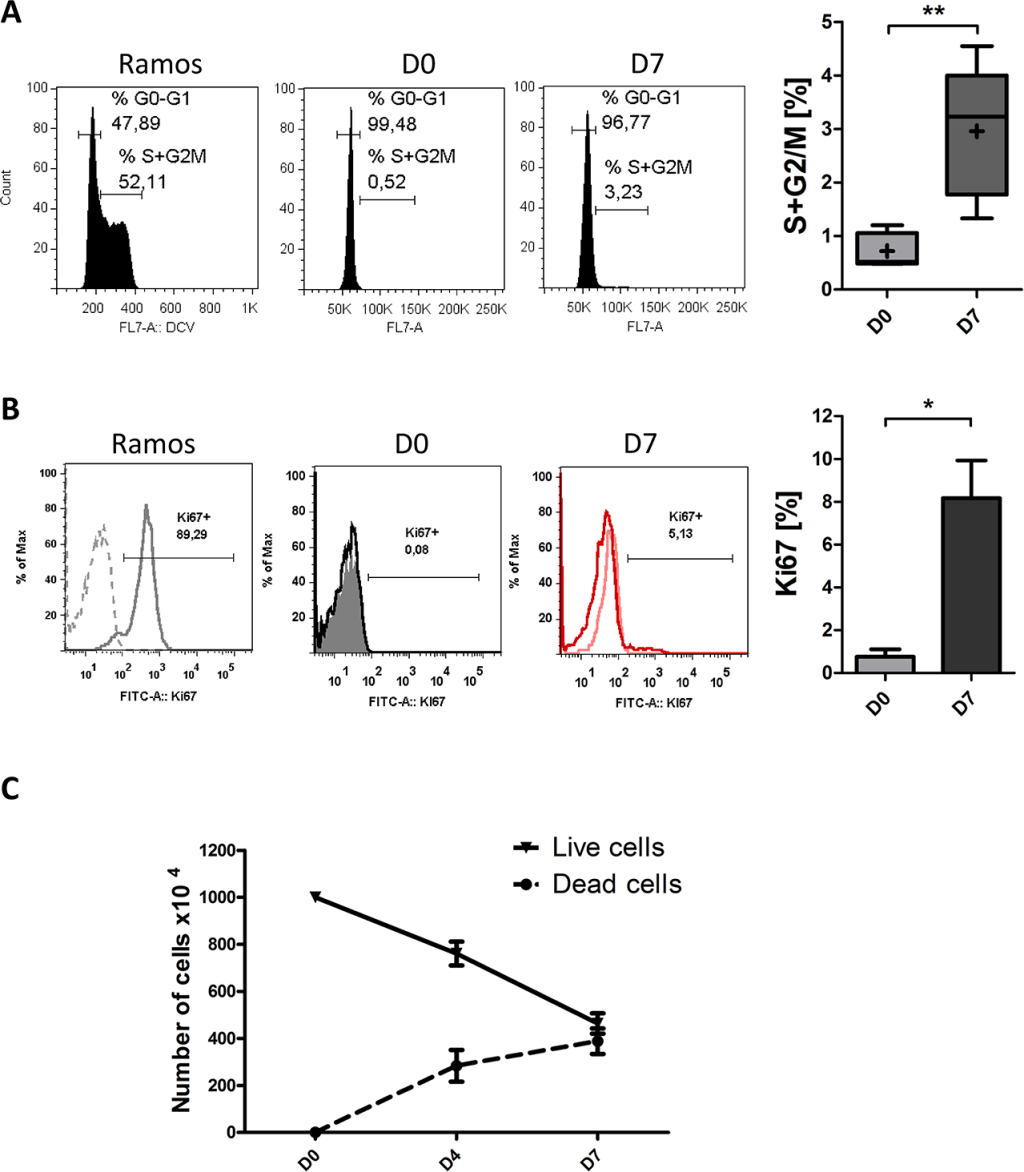
Differentiated CLL B-cells display a low proliferation rate **A.** At D0 and D7 of culture, the DNA content of living cells was measured by DyeCycle Violet staining. Results are represented as the summed percentages of cells in the S and G2/M phases of the cell cycle. Cytometry plots are representative of the results from five experiments. Ramos cell line (growing in the log phase) was used as control. Day 7 values were compared with D0 values and statistical significance was calculated using Wilcoxon's test: **p < 0.01. **B.** At D0 and D7, cells were labeled after permeabilization with FITC-conjugated anti-Ki67 mAbs or isotype-control mAbs. Cytometry plots are representative of the results from three experiments. Ramos cell line, growing in log phase, was used as control. Significance was calculated using a paired *t*-test: *p < 0.05. **C.** Live and dead cells were counted at the indicated times in a trypan blue dye exclusion assay. The results are representative of eleven experiments.

In contrast to the majority of human tumors, CLL B-cells are arrested in the G0-G1 phase of the cell cycle [21]. We therefore examined the *in vitro* cell cycle distribution of the generated ASCs and the expression of Ki67. Our analysis revealed a significant increase in cycling cells between D0 and D7, however, the mean percentage of cycling cells on D7 itself was remarkably low (3 ± 1.2%) (Figure 6A). These results correlated with those obtained with Ki67 staining that show a percentage of 8 ± 3% of Ki67-positive cells at D7 (Figure 6B). These cycling cells might have downregulated p27 and upregulated survivin [22, 40, 48]. However, this low percentage of cycling cells on D7 could be explained by (i) the repression by BLIMP1 of factors associated with cell cycle and BCR signaling, such as BCL6, c-MYC and BTK [40, 41, 53] and (ii) the fact that cell cycle entry is rendered irrelevant by cell death due to the differentiation. Nevertheless, cell death and proliferation were also assessed in a trypan blue assay at D0, D4 and D7. As shown in Figure 6C, differentiation was associated with a decrease in the viable cell count and an increase in the dead cell count. Taken as a whole, our findings suggest that the differentiation of CLL B-cells into ASCs is associated with incidence of apoptosis but not exaggerated cell proliferation.

### 5- CpG/CD40L/c-derived CLL B-cells differentiation induces changes that are similar to those observed in PMA/CD40L/c-derived differentiation

**Figure 7:**
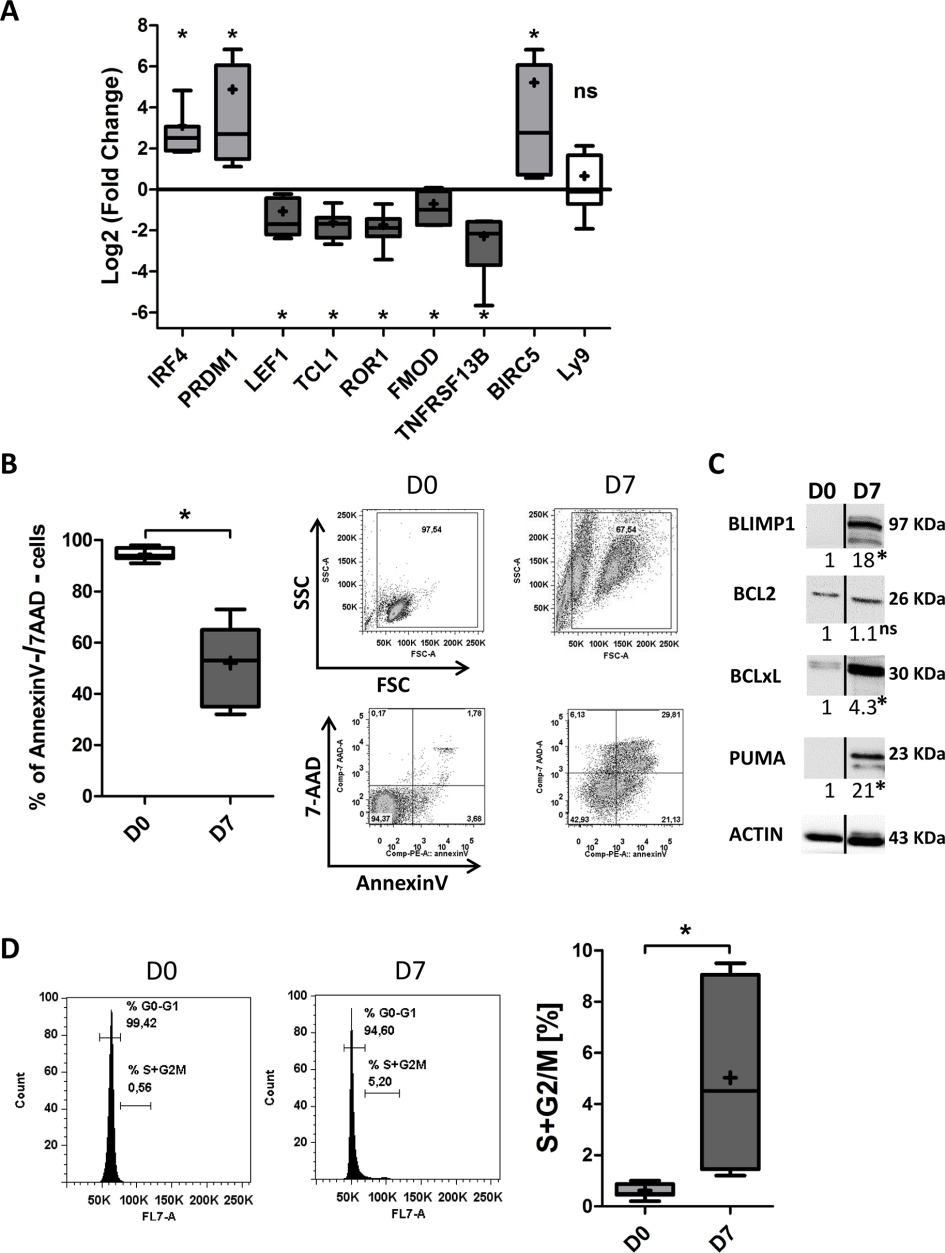
CpG/CD40L/c-derived CLL B-cells differentiation induces downregulation of the expression of CLL-pathogenesis-associated factors, decreased survival and a low proliferation rate On D0, CLL B-cells were stimulated with CpG and CD40L, in combination with the cytokines IL-2, IL-10 and IL-15. On D4, cells were harvested and incubated with IL-2, IL-6, IL-10 and IL-15 for 3 days. **A.** The expression of the IRF4, PRDM1, LEF1, TCL1, ROR1, FMOD, TNFRSF13B, BIRC5 and Ly9 genes was evaluated by quantitative real-time RT-PCR on D0 and D7. Results are expressed relative to gene expression in CLL B-cells on D0, according to the 2^−ΔΔCT^ method. The results are represented as log2 fold changes in box-and-whisker (min to max) plots (the “+” sign indicates the mean) for seven experiments. Statistical significance was calculated using the Wilcoxon's test: *p < 0.05, ns, not significant. **B.** Cells were stained with Annexin V-PE and 7AAD at D0 and D7 to evaluate apoptosis. Left panel: the percentages of double-negative (i.e. annexin-V-negative and 7AAD-negative) living cells in seven experiments are represented in box-and-whisker (min to max) plots (the “+” sign indicates the mean). Right panel: cytometry plots from a representative patient. Statistical significance was calculated using the Wilcoxon's test: *p < 0.05. **C.** Immunoblot analysis and densitometry values of BLIMP1, BCL2, BCLxL, and PUMA in cells at D0 and D7. The PUMA antibody also cross-reacts with an 18 kDa band of unknown origin. The black dividing lines on the blot data indicate that lanes are run on different parts of the same gel (non-adjacent lanes). The data shown are representative of three experiments. Statistical significance was calculated using Student's *t*-test: *p < 0.05. **D.** At D0 and D7 of culture, the DNA content of living cells was measured by DyeCycle Violet staining. Results are represented as the summed percentages of cells in the S and G2/M phases of the cell cycle. Cytometry plots are representative of the results from five experiments. Day 7 values were compared with D0 values and statistical significance was calculated using Wilcoxon's test: *p < 0.05.

In order to establish whether we would obtain the same effects on CLL-pathogenesis-associated factors with other differentiation-promoting agents, we replaced PMA by CpG oligodeoxynucleotide. Quantitative RT-PCRs showed that the differentiation of CLL B-cells into ASCs induced significant downregulation of LEF1 (5.3-fold), TCL1 (8.2-fold), ROR1 (7-fold), TNFRSF13B/TACI (8-fold) and FMOD (3.9-fold) (Figure 7A), and significant upregulation of BIRC5/survivin (36-fold) (Figure 7A). However, there was no significant effect on the expression of Ly9 (Figure 7A). An annexin-V/7AAD survival assay detected apoptosis among the generated ASCs (on average, 52 ± 16% of the cells had survived; Figure 7B). Western blot analysis showed no changes in the expression of BCL2 and upregulation of BCLxL and PUMA (4.2-fold and 21.7-fold, respectively) (Figure 7C). An analysis of the cell cycle distribution revealed a significant increase in cycling cells between D0 and D7, although the mean percentage of cycling cells on D7 itself was relatively low (5 ± 3.6%) (Figure 7D). These results are in agreement with those obtained with the PMA/CD40L/c system and suggest that the observed changes in the expression of CLL-pathogenesis-associated factors are indeed related to differentiation.

## DISCUSSION

The concept whereby malignant B-cells are induced to differentiate into a more mature, non-malignant or less malignant state is clinically plausible and can be a promising strategy as differentiation therapy in CLL [14, 29, 33–35]. To the best of our knowledge, the present report is the first to demonstrate the modulatory effects of differentiation on factors that have an important role in physiopathology of CLL, including LEF1, TCL1, ROR1, FMOD, *TNFRSF13B*, PI3K, BTK, p27, BCL2, BCLXL, PUMA and MCL1. Many of these factors distinguish CLL B-cells from normal mature B-cells and represent a significant proportion of the malignant program in CLL B-cells. Here, we showed that differentiation of CLL B-cells into ASCs leads to decreased expression of these factors - suggesting that restoring the terminal differentiation program in CLL B-cells may lead to the suppression of their malignant program. The resulting ASCs might be less malignant or non-malignant, and would thus fail to sustain malignant growth. Importantly, differentiation of CLL B-cells into ASCs was associated with a decrease in cell survival but not with massive cell proliferation - suggesting that differentiation might be an effective therapy for this mature B-cell malignancy. However, future research should focus on the leukemogenicity and pathogenicity of the generated ASCs in animal models and should establish whether these cells are no longer able to cause disease.

In addition to differentiation-dependent apoptosis, differentiation therapy in CLL could potentially be combined with other targeted therapies or immunotherapy [35]. Indeed, terminal differentiation confers exquisite apoptotic sensitivity to proteasome inhibitors, inhibitors of the ER stress-associated pathway (IRE1/XBP1) [59, 60], inhibitors of HSP90 [61], BCL2, BCLxL (e.g. ABT-199 and ABT-737) [3, 4] and survivin [55]. As we have shown here and in recent work [34], levels of these targets (e.g. BCLxL and survivin) are exacerbated by terminal differentiation of leukemic cells; indeed, a number of the corresponding inhibitors appear to have potential as treatments for CLL [3, 4, 55, 59–61]. It may be of value to target the disruption of the fragile balance between pro- and anti-apoptotic proteins that occurs during differentiation. Given the central role of BCLxL in this balance, we speculate that differentiation will sensitize cells to BCLxL inhibitors [4, 57]. Furthermore, the cellular and molecular microenvironment (manipulated by leukemic cells themselves) confers a selective advantage on CLL B-cells and enables disease progression. CLL pathogenesis, survival, progression and resistance to therapy are influenced by microenvironmental stimuli such as BCR ligation, cell-cell interaction and soluble factor [22, 25, 62]. The changes in intra- and extracellular signaling pathways induced by differentiation of CLL B-cells might restrict the latter's dependency on their microenvironment and deprive them of survival and growth stimuli. Thus, the downregulation of CXCR4 and TACI induced by differentiation of CLL B-cells may deprive the cells of survival mediators including the TACI ligands BAFF and APRIL and the CXCR4 ligand CXCL12 [48, 57]. We speculate that in CLL, differentiation therapy would have the advantage of inducing direct changes in CLL B-cells; this would increase their sensitivity to death signals, render them less dependent on their microenvironment and enhance their sensitivity to targeted or immuno-therapies.

Proliferation and apoptosis process are involved in the differentiation of B-cells into ASCs [63]. Plasma cell differentiation requires the regulation of proliferation and is probably associated with irrevocable cell cycle exit [53]. Indeed, it is questionable whether plasma cell differentiation can occur in the absence of cell division [53]. We think that cells may need to divide at least once before they can differentiate into ASCs. Passage through the cell cycle will probably enable the molecular and epigenetic modifications required for differentiation [53, 63]. Indeed, cell cycle entry in our culture (3 ± 1.2% for PMA/CD40L/c system, 5 ± 3.6% for CpG/CD40L/c system) is very low in comparison with that observed for differentiating cells in a normal human B-cell differentiation system (between 15% and 35% in S-phase) [41, 64–67]. However, as pointed out above, it will be important to study the leukemogenicity and pathogenicity of these cells in an animal model.

Our culture system is not optimized for clinical use. In particular, our culture method is constrained by its two-step configuration and the varied number of cytokines used. We are in an *in vitro* context; we were mainly concerning about finding optimal differentiation conditions. Our differentiation model was based on terminal differentiation culture systems for normal B-cells guaranteeing optimal differentiation conditions [41, 64–68]. Nevertheless, the CLL microenvironment includes CD40L (from activated T-cells) and microenvironment-derived cytokines (secreted by dendritic cells, T-cells, stroma cells and nurse-like cells) [69, 70]. Moreover, the differentiation of CLL cells into ACSs has been shown to occur spontaneously *in vivo* [71–74]. Furthermore, stimulation of CLL B-cells with CpG was shown to induce autocrine IL-6 and IL-10 production [75]. Exposure to these factors and a differentiation-promoting agent might create a favorable environment for the terminal differentiation of CLL B-cells *in vivo*. Recent studies have identified critical role for IL-21 in terminal human B-cells differentiation into ASC. The effect of IL-21 on terminal B-cell differentiation was found to exceed that of IL-2, IL-4, IL-13, and IL-10 by up to 100-fold [76]. However, the effect of IL-21 could be potentiated by these cytokines [76]. Moreover, very recently it was shown that CpG and IL-21 are interesting differentiation-promoting agents in CLL cells [14, 33]. There is a large body of research in favor of TLR9-targeted therapy for CLL [77, 78]. The TRL9-ligand CpG induces the differentiation and apoptosis of CLL B-cells [14, 33, 35, 75]. Indeed, in agreement with our results, Gutierrez [14] have shown that CLL B-cells induced to differentiate into ASCs by CpG show low levels of LEF1 expression and decreased activation of Wnt pathway. LEF1 and ROR1 are important effectors of the Wnt/β-catenin signaling pathway that controls cell growth, survival and differentiation [11, 16]. Indeed, LEF1 and ROR1 are expressed by a variety of human cancers including melanoma, colorectal cancer, pancreatic cancer and lung cancer [11, 12, 79]. Importantly, we and others [35, 78, 80] have shown that CpG treatment of CLL B-cells induces the upregulation of CD20 expression. We speculate that CpG treatment can increase the sensitivity of CLL cells to anti-CD20 therapy. Sagiv-Barfi et al [81] very recently developed an interesting approach for treating lymphoma in mouse model by combining active immunotherapy and targeted kinase inhibition. Injection of intratumoral CpG and systemic treatment with ibrutinib resulted in the full, permanent regression of both local and distant tumors. Phorbol myristate acetate is a polyclonal activator of normal B-cells and CLL B-cells [34, 82]. Our unpublished data and previously published data [82] show that PMA has a specific differentiation effect on CLL B-cells and has no effect on other B-cell malignancies. PMA activates the PKC pathway by mimicking diacylglycerol (a natural PKC ligand and activator) [83, 84]. The mediation, by PKC, of the PMA-dependent activation and differentiation of B-cells has been demonstrated in experiments using an inhibitor of PKC [85, 86]. Phorbol ester has been proposed and tested as potential therapeutic agent in pre-clinical and clinical models [32, 84, 87, 88]. Indeed, studies in patients with hematological malignancies evidenced the feasibility of PMA administration resulting in therapeutic responses [32, 88]. The role of PKC in inducing CLL B-cells differentiation was also demonstrated with another activator of the PKC pathway “bryostatin” which lack carcinogenic potential [39, 89]. Clinical trials have shown that bryostatin has moderate activity as a single agent or when combined with fludarabine in the treatment of CLL [39, 90]. Given that levels of some target molecules (BCL-XL, survivin, and factors in the ER stress-associated pathway) increase during the differentiation process of CLL B-cells, we speculate that bryostatin might be usefully combined with the corresponding inhibitors of these molecules (ABT-737 [3], YM155 [55] and B-I09 [59]).

CLL is characterized by an important immunological dysfunction including immunoglobulin production. Over 60% of patients develop hypogammaglobulinaemia during the course of CLL, leading to recurrent infections (the most common cause of death in this disease) [91]. Moreover, low levels of immunoglobulin and complement may decrease the clearance of auto antigens (apoptotic antigens), with the subsequently increased risk of autoimmunity. Indeed, in nine out of eleven patients, serum IgM levels were below the normal range (Table 1). One can reasonably hypothesize that differentiation of CLL B-cells into ASCs will be a useful way of restoring levels of Ig (IgM, at least) in CLL. However, as we and other have shown [34, 35, 92], in some cases of CLL, the IgMs produced may display auto/polyreactivity and thus may induce autoimmune disease. Importantly, pathogenic autoantibodies in CLL are polyclonal and seem to be produced by residual nonmalignant B-cells [93]. Nevertheless, the affinity of antibodies might be too low to trigger an autoimmune response; rather, the antibodies produced might bind to invading pathogens and provide a first line of humoral defense against infection and/or might be involved in various homeostatic functions (clearance of apoptotic cells and tumor cells), acting as natural antibodies [92, 94]. Indeed, it has been shown that CLL BCRs bind to apoptotic antigens as well as antigenic determinants of bacterial capsules and toxins or viral coats and fungi [92, 94–96].

**Table 1:** Patient characteristics

Patient	sex	age	Binetstage	Matutes score	CD38	Cytogenetics	mutational status	Serum IgMg/l (Normal range 0, 45–1, 5 g/l)
1	M	80	A	5	-	NORMAL	UM	0, 19
2	M	56	A	5	-	13q14 del	M (8.3%)	0, 8
3	F	67	A	4	-	ND	M (10.6%)	0, 31
4	M	68	A	5	-	17p del	UM	ND
5	M	64	A	5	-	Trisomy 12	UM	0, 4
6	M	76	A	5	-	Trisomy 12	ND	0, 37
7	M	82	A	5	-	NORMAL	M (8.3%)	0, 17
8	F	57	A	4	-	ND	ND	0, 17
9	M	63	A	5	-	13q14 del	M (10.7%)	ND
10	F	81	A	5	-	ND	UM	0, 29
11	M	48	B	5	-	13q14, 11q del	UM	ND
12	M	67	B	5	-	13q14 del	UM	0, 44
13	F	77	A	5	-	NORMAL	ND	0, 33
14	M	52	A	4	-	ND	UM	ND
15	M	76	B	5	-	13q14, 11q del	ND	0, 21

del, deletion; ND, not determined; M, mutated; UN, unmutated

**Figure 8:**
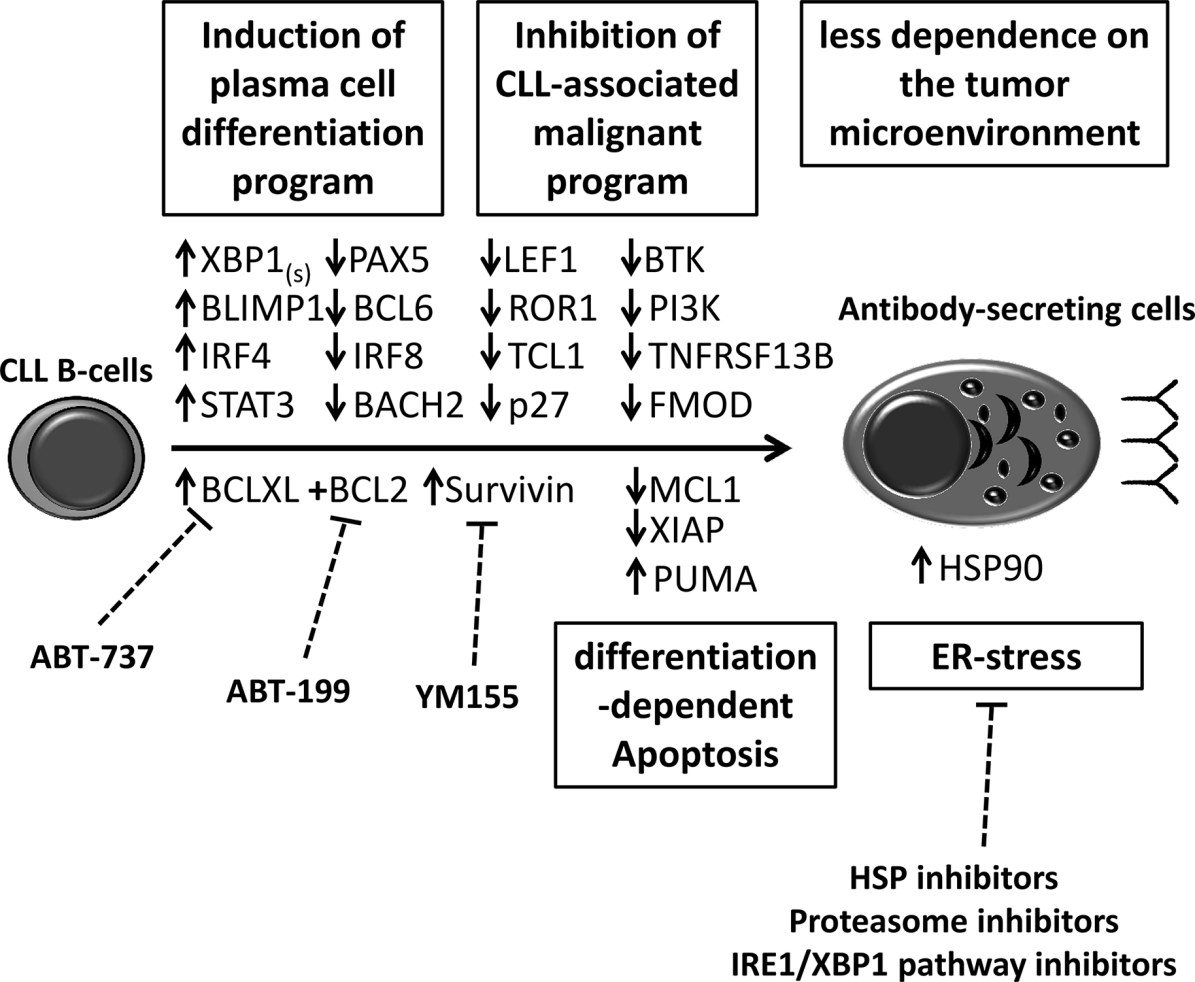
Differentiation therapy would have the advantage of inducing direct changes in CLL B-cells Differentiation of CLL B-cells into antibody-secreting cells leads to depletion of malignant program and deregulation of the apoptosis/survival balance. Differentiation of CLL B-cells may facilitate sensitivity towards targeted therapy such as BCL2 family inhibitors (ABT-737 and ABT-199), survivin inhibitors (YM155) and heat shock protein (HSP) inhibitors.

Lastly, the suppression of expression of the malignant program and the deregulation of the apoptosis/survival balance observed during the terminal differentiation of CLL B-cells emphasizes that differentiation therapy might be effective in CLL (Figure 8). Furthermore, analysis of the molecular mechanisms during CLL B-cells differentiation might provide selective and targeted molecules for novel treatment strategies (Figure 8). Our findings [34, 35] and those of others [14, 33, 36] form a rational basis for the further development of differentiation therapy in CLL. This approach should be facilitated by the availability of interesting agents (such as CpG) [14, 35] but above all by (i) the identification of novel and safe agents promoting B-cell differentiating (e.g. epigenetic modifiers [33]), (ii) the development of technologies and strategies allowing selective targeting of leukemic cells [97] and (iii) the development of an *in vivo* animal model.

## MATERIALS AND METHODS

### Patients

Chronic lymphocytic leukemia B-cells were obtained from the peripheral blood of 11 untreated patients having been diagnosed in accordance with international guidelines (Table 1). All patients provided their written, informed consent to participate in the study. All procedures involving samples from patients were approved by the local institutional review board (Comité de Protection des Personnes Nord-Ouest, Amiens, France).

### Immunophenotypic analysis

Cells were stained with the appropriate combinations of fluorochrome-conjugated Abs, in a three- to five-color direct immunofluorescence staining protocol. All antibodies were purchased from BD Biosciences (Le Pont de Claix, France). The Cytofix/Cytoperm kit (BD Biosciences) was used for the intracellular staining of immunoglobulin M (IgM) and Ki67, according to the manufacturer's recommendations. Flow cytometry analysis was performed with a FACSCantoII flow cytometer (BD Biosciences). FlowJo software (Tree Star, Ashland, OR, USA) was used for data analysis.

### CLL B-cell purification and culture

Peripheral blood mononuclear cells were isolated by Ficoll density gradient centrifugation of heparinized venous blood samples from CLL patients. CD19+CD5+ CLL B-cells were purified by negative selection using magnetic bead-activated cell sorting (MACS), with a B cell (B-CLL) isolation kit (Miltenyi Biotec). The purity of all preparations was around 98% and the cells co-expressed CD19 and CD5 at their surface (as assessed by flow cytometry). Direct labeling with anti-CD2, CD14 and CD56 antibodies was always used to check that purified CLL B-cells were not contaminated by other immune cells. On day (D) 0, purified CLL B-cells were seeded at a concentration of 2 × 10^6^/ml and stimulated for four days with PMA (1 μg/ml, Santa Cruz Biotechnology, Heidelberg, Germany) or with phosphorothioate CpG oligodeoxynucleotide 2006 (10 μg/ml; Sigma-Aldrich) in association with histidine-tagged soluble recombinant human CD40L (50 ng/ml), anti-polyhistidine monoclonal antibody (mAb) (5 μg/ml; R&D Systems, Abingdon, UK) and interleukins (IL)-2 (50 ng/ml), IL-10 (50 ng/ml) and IL-15 (10 ng/ml). The cells were cultured in 5 ml wells in six-well, flat-bottomed culture plates. On D4, the cells were harvested, washed and seeded at a concentration of 10^6^/ml in the presence of IL-2 (50 ng/ml), IL-6 (50 ng/ml), IL-10 (50 ng/ml), and IL-15 (10 ng/ml) for 3 days. On D7, cells were harvested, washed and analyzed. All human recombinant cytokines were purchased from PeproTech EC (Neuilly-Sur-Seine, France).

### Quantitative real-time-PCR (qRT-PCR) analysis

The qRT-PCR analysis was performed on a StepOnePlus™ Real-time PCR System (Applied Biosystems, Courtaboeuf, France) as previously described. [34] The TaqMan Gene Expression assays for PRDM1 (BLIMP1) (assay ID Hs00153357_m1), IRF4 (Hs01056533_m1), XBP1s (Hs03929085_g1), PAX5 (Hs00172003_m1), BCL6 (Hs00277037_m1), IRF8 (Hs01128710_m1), BACH2 (Hs00222364_m1), BATF (Hs00232390_m1), GAS6 (Hs01090305_m1), CD138 (Hs00896423_m1), LEF1 (Hs01547250_m1), TCL1A (Hs00951350_m1), ROR1 (Hs00938677_m1), FCER2 (Hs00233627_m1), BIRC5 (HS04194392_s1), FMOD (Hs00157619_m1), MARCKS (Hs00158993_m1), ATXN1 (Hs00165656_m1), Ly9 (Hs03004330_m1), TNFRSF13B (Hs00963364_m1) and EBF1 (Hs00395524_m1) were purchased from Applied Biosystems.

### Immunoblotting

Western blotting was performed as previously described [34], with antibodies against CD229, c-MYC, HSP90 and actin (from Santa Cruz Biotechnology Inc.), LEF1, ROR1, p27, PI3K, BTK, BCL2, BLIMP1, IRF4, survivin, TYK2, STAT3, XIAP, MCL1, PUMA and BCLxL (from Cell Signaling Technology, Danvers, MA, USA) and FMOD (from Sigma-Aldrich, France). The anti-survivin antibody used in our work does not detect survivin splicing forms. The results were visualized on a ChemiDocTM MP Imaging System (Bio-Rad, Marnes-la-Coquette, France). Densitometric quantification was performed with ImageJ analysis software (NIH) (http://rsbweb.nih.gov/ij/).

### Cell viability and cell cycle analysis

Cell viability was measured by flow cytometry using annexin-V-phycoerythrin (PE) and 7-amino-actinomycin (7-AAD) staining kit (BD Biosciences) according to the manufacturer's recommendations. Cell cycle status was assessed using Vybrant DyeCycle Violet stain (Invitrogen, Courtaboeuf, France), according to the manufacturer's instructions. Briefly, 3 × 10^5^ cells were suspended in complete medium containing 0.5 μl of Vybrant DyeCycle Violet stain for 30 minutes at 37°C.

Cells were analyzed with a FACSCanto flow cytometer (BD Biosciences) and data analysis was performed by FlowJo software (Tree Star).

### Analysis of IgM, IgG and IgA secretion

The levels of human IgM, IgG, and IgA in the culture supernatants were quantified with the corresponding ELISA kit (Bethyl Laboratories, Montgomery, TX, USA).

### Statistical analysis

All statistical analyses were performed with Prism 5 software (GraphPad Software, La Jolla, CA, USA). The statistical significance was determined using Wilcoxon's test or Student's *t*-test, as appropriate. *p* values < 0.05 were considered to be statistically significant. Differences are denoted as follows: *p < 0.05, **p < 0.01 and ***p < 0.001.

## SUPPLEMENTARY FIGURES


