# Supplementary material for: Factors involved in CLL pathogenesis and cell survival are disrupted by differentiation of CLL B-cells into antibody-secreting cells
Source: Oncotarget. 2015 May 11;6(21):18484–503. doi: 10.18632/oncotarget.3941 (PMC4621905; doi:10.18632/oncotarget.3941)
Supplement: Supplementary file 1 [file oncotarget-06-18484-s001.pdf]

SUPPLEMENTARY FIGURES

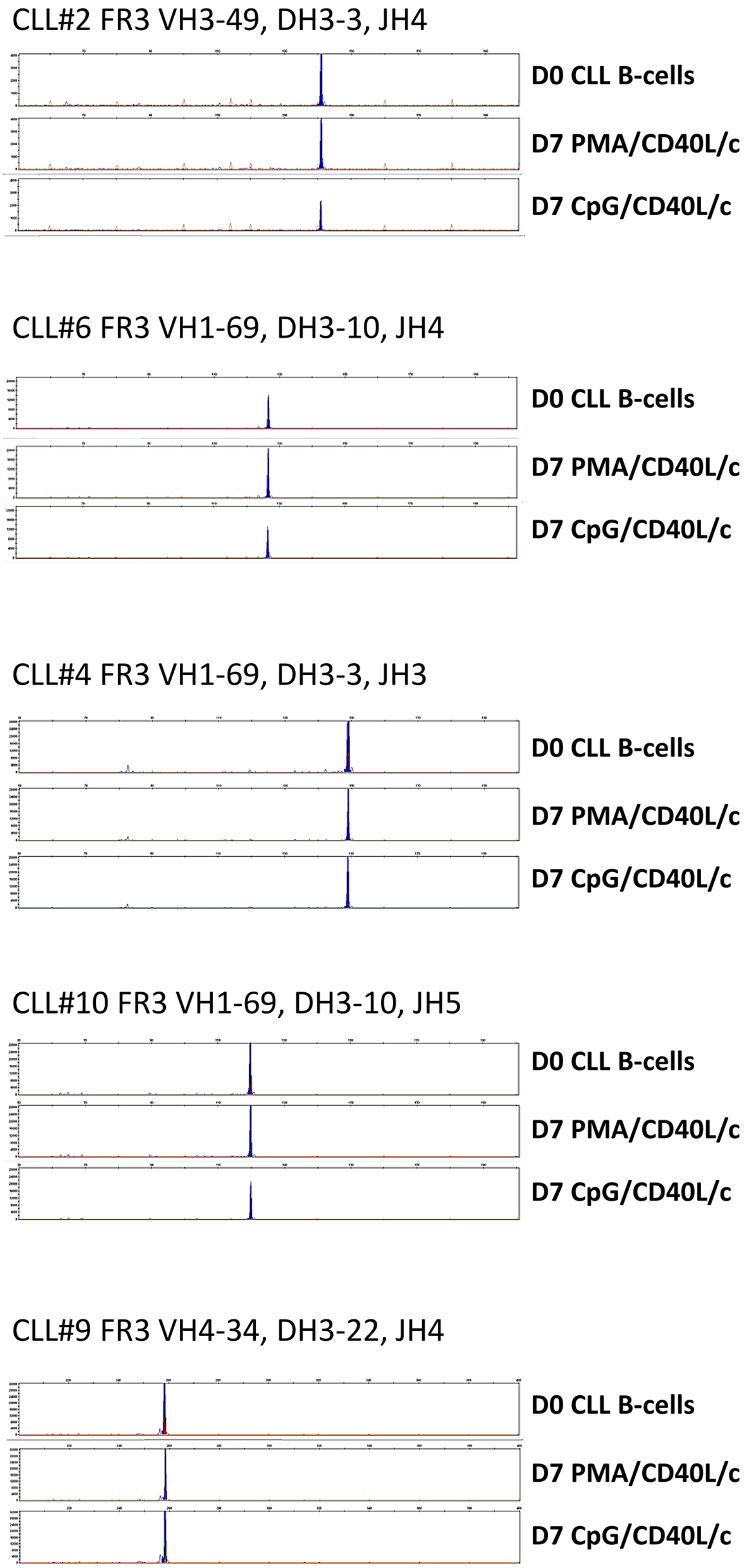

Supplementary Figure S1: GeneScan analysis of IgH gene rearrangements shows a monoclonal pattern at D0 and D7. Fragments are aligned by size (indicated above and at the bottom of each panel). Left panels show a peak in the IgH framework 3 (FR3)-specific size range for D0 CLL B-cells (upper panel), D7 PMA/CD40L/c-stimulated cells (middle panel) and D7 CpG/CD40L/c-stimulated cells (lower panel), indicating a monoclonal rearrangement.

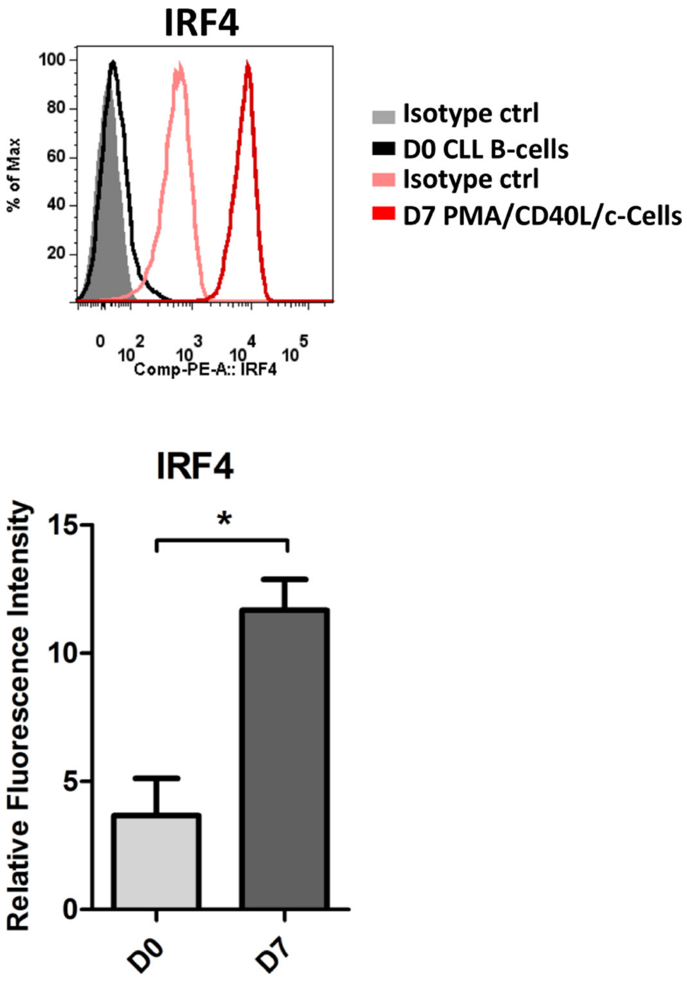

Supplementary Figure S2: Upregulation of IRF4 expression in D7 ASCs. Cells were labeled at D0 and D7 with anti-IRF4 mAbs after permeabilization. Upper panel: cytometry plots for a representative patient. Lower panel: relative fluorescence intensity (RFI) were calculated as the ratio of the MFI of cells labeled with a specific Ab to that of cells labeled with a matched isotype control. Bars represent RFI mean values±SEM in three experiments. Statistical significance was calculated using a paired *t*-test: *$p < 0.05$.

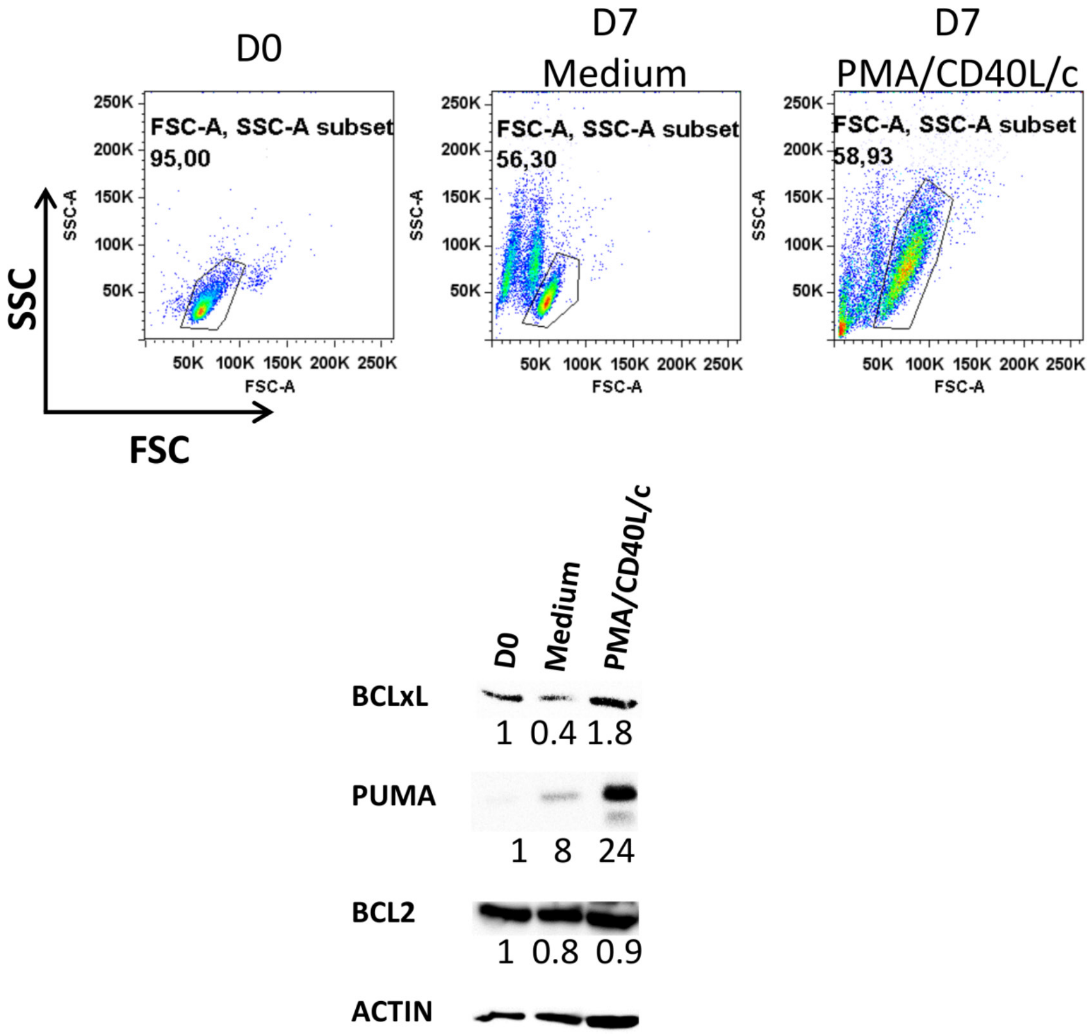

Supplementary Figure S3: Upregulation of BCLxL and PUMA in D7 ASCs but not in unstimulated cells. Upper panel: side scatter (SSC)/forward scatter (FCS) dot plot of cells analyzed by immunoblotting. Lower panel: Immunoblot analysis and densitometry values for BCL2, BCLxL and PUMA in cells at D0, unstimulated cells at D7 (medium only) and stimulated cells at D7 (PMA/CD40L/c). The data are representative of two experiments.
